# Dual-energy CT in the diagnosis of occult acute scaphoid injury: a direct comparison with MRI

**DOI:** 10.1007/s00330-020-07604-z

**Published:** 2020-12-19

**Authors:** Cheng Xie, Sarim Ather, Ramy Mansour, Fergus Gleeson, Rajat Chowdhury

**Affiliations:** 1 Department of Radiology, John Radcliffe Hospital, Oxford University Hospitals NHS Foundation Trust, Headley Way, Headington, OX3 9DU Oxford, UK; 2 Department of Radiology, Nuffield Orthopaedic Centre, Oxford University Hospitals NHS Foundation Trust, Windmill Road, Headington, OX3 7HE Oxford, UK; 3 Department of Radiology, Churchill Hospital, Oxford University Hospitals NHS Foundation Trust, Old Road, Headington, OX3 7LE Oxford, UK

**Keywords:** Wrist injuries, Scaphoid bone, Bone marrow, Edema, Radiography

## Abstract

**Objectives:**

Scaphoid injuries occult on plain radiography often require further imaging for definitive diagnosis. We investigate the utility of dual-energy computed tomography (DECT) for the detection of acute bone marrow oedema and fracture of scaphoid compared to MRI.

**Materials and methods:**

Twenty patients who presented acutely (without prior injury) to the emergency department with clinically suspected occult scaphoid fracture and had MRI of the wrist were prospectively recruited to have DECT (GE Revolution CT). Material decomposition images of the water-calcium base pair were generated and assessed in conjunction with the monochromatic images to permit correlation of marrow signal changes with any cortical disruption for fracture confirmation. The assessment was performed by two musculoskeletal radiologists blinded from MRI results. The statistical difference of MRI and reviewers’ detection of acute bone oedema (1 = present, 0 = absent) was performed using the Friedman test (SPSS v.16).

**Results:**

MRI showed acute scaphoid fracture and/or bone marrow oedema in 14/20 patients of which 6 also had cortical disruption. On DECT, reviewer A identified oedema in 13 and cortical disruption in 10 patients while reviewer B identified oedema in 10 and cortical disruption in seven of the 14 MRI positive patients. No statistically significant difference in oedema detection on MRI and reviewers of DECT (*p* value 0.61) but DECT was more sensitive at detecting cortical disruption.

**Conclusion:**

DECT has the capability to detect acute scaphoid oedema in addition to cortical fractures. However, compared to MRI, DECT has lower contrast resolution and less sensitive in the detection of mild oedema.

**Key Points:**

*• Dual-energy CT is able to detect acute traumatic scaphoid marrow oedema.*

*• Dual-energy CT has greater detection rate of scaphoid fractures than MRI.*

*• Dual-energy CT is an alternative to MRI for occult scaphoid injury.*

**Supplementary Information:**

The online version contains supplementary material available at 10.1007/s00330-020-07604-z.

## Introduction

After a fall on to an outstretched hand (FOOSH), the scaphoid is the most frequently injured carpal bone and represents 2–3% of all fractures [1]. A timely diagnosis is important to reduce the risk of non-union, avascular necrosis, and subsequent comorbidities. When there is a convincing mechanism of injury and clinical findings (anatomical snuffbox tenderness and/or pain on axial compression of the thumb), a plain radiograph is usually the initial step in the imaging workup. Plain radiography will detect the majority of scaphoid fractures. However, occult scaphoid fractures that are not detectable on the initial plain radiograph constitute up to 20% of scaphoid injuries [2]. At this stage, a number of second-line imaging options are available. The inconsistencies in the next line of investigation can lead to a delay in diagnosis and unnecessary treatment.

Currently, there is no consensus on the best second-line imaging modality to detect occult scaphoid fractures. The different diagnostic pathways after the patients’ first presentation can be a clinical follow-up and imaging with a repeat plain film, MRI, CT, or bone scan [3]. The many options available can further increase the time taken to exclude an occult scaphoid fracture.

At our institution, there is a high dependence on magnetic resonance imaging (MRI) to exclude the occult scaphoid fractures; however, due to limited availability, patients are usually unable to get a definitive diagnosis on the day of the injury. Studies in this area have shown that DECT has comparable results to MRI in identifying fractures and acute bone marrow oedema of the ankle, knee, hip, spine, and calcaneus [4–8]. A recent study and case report that focused on acute scaphoid bone marrow oedema also supports the use of DECT as an alternative to MRI [9, 10]. To our knowledge, all the current published work on DECT detection of bone marrow oedema has been conducted using the Siemens dual-source scanner and the virtual non-calcium (VNC) tool. Our study would be the first to investigate the use of GE Revolution CT, a single-source, rapid voltage switching DECT, and the water-calcium material decomposition images for this purpose. The objective of this study is to investigate the use of this specific DECT design in the detection of occult acute scaphoid fractures and associated bone marrow oedema in direct comparison with MRI.

## Materials and methods

Ethical approval was obtained for this prospective study to investigate the use of DECT in patients, who have a clinically suspected scaphoid fracture, with direct correlation to their MRI. A total of 20 patients were recruited for this study.

## Patient recruitment

Patients who present acutely to the emergency department with negative findings on plain radiograph but suspected to have an occult scaphoid fracture and referred for an MRI scan were recruited for this study. The patients’ MRI took place normally as per institution protocol, and all these patients were given information leaflets about the study after the MRI scan. The MRI scans were obtained with a 3-T HDx 750 (GE Medical Systems). The protocol included coronal T1 (slice thickness = 3 mm with 3 mm spacing, TR = 580 ms, TE = 12 ms, flip angle = 150), coronal STIR (slice thickness = 3 mm with 3-mm spacing, TR = 3000 ms, TE = 60 ms, flip angle = 150) and axial proton density fat-saturated sequences (slice thickness = 3 mm with 3 mm spacing, TR = 4400 ms, TE = 30 ms, flip angle = 150).

When the patients attended for their MRI scan, those who met the eligibility criteria (Table 1) were recruited by one of the study investigators. Formal written consent was taken from patients who agreed to take part in the study. Supplementary Figure 1 demonstrates the study flow and the usual patient pathway.

**Table 1:** Eligibility criteria

Inclusion criteria	Exclusion criteria
Adult patients (age 18 and over)	Patients with scaphoid fractures identified on plain film
Males and females	Mechanism of injury unknown
Clear mechanism of injury and clinically suspected acute scaphoid fracture	Previous scaphoid fracture to the injured wrist
Negative findings on initial plain radiograph for acute scaphoid fracture	Chronic inflammatory joint conditions – rheumatoid arthritis or connective tissue disorders
Patient is scheduled for a MRI examination.	Suspicion of infective cause (ie septic arthritis)
	Extremely frail patients
	Pregnant patients
	Patients who are unable to provide informed consent

## Image acquisition

DECT imaging was performed on a Revolution CT (GE Medical Systems), which uses a single x-ray source with rapid asynchronous switching of the energy levels to acquire high- and low-kVp images in an interleaved fashion and a single detector that registers information from both energies. The images for the current study were obtained using the following scan parameters: helical, rapid switch between tube voltages of 70 and 140 kVp in 0.5 ms; tube current, 260 mA; rotation time = 0.69 s; slice thickness, 0.625 mm; and slice interval, 0.625 mm.

### Image post-processing

Image post-processing was carried out using the Gemstone Spectral Imaging (GSI) viewer software on an Advanced Workstation Server 2.0 (AW 2.0; GE Healthcare). The high- and low-energy view data were processed using a projection-based material decomposition algorithm to generate material density images for the water-calcium base pairs. A colour overlay was added to the material density images for visual inspection. In addition, monochromatic images at 0.625-mm slice thickness and interval were also generated for fracture assessment using bone and soft-tissue kernels.

### Image analysis

Qualitative assessment of the DECT images of the 20 participants was carried out by two experienced musculoskeletal consultant radiologists blinded to the MRI findings. They analysed the monochromatic images for the presence of cortical disruption and the material density images for the presence of bone marrow oedema.

Another musculoskeletal consultant radiologist reviewed the MRI images of the 20 participants for the presence of fracture and marrow oedema.

A study investigator not part of the imaging analysis process performed the data analysis. The statistical difference of the MRI against reviewers’ detection of acute bone oedema (1 = present, 0 = absent) was performed using the Friedman test (SPSS v.16). Statistical significance is taken as *p* value < 0.05.

## Results

A total of 20 patients were recruited for the study. This included 12 male and eight female patients, with a median age of 51 years (range, 20–76 years).

Supplementary Table 1 summarises the study findings including time from injury to presentation and imaging, the presence of scaphoid injury on MRI and DECT (for each reviewer independently), presence of non-scaphoid bony and soft-tissue injuries, and patient management.

The median time from the date of injury to the MRI scan was 13.5 days (range, 3–51 days). The median time from MRI to DECT was 6 days (range, 0–9 days) and 18 days from the date of injury (range, 6–57 days).

The MRI examination showed scaphoid bone marrow oedema in 14 of the 20 patients with corresponding fractures in six of the 14 patients. Other bony injuries were demonstrated in six patients and ligamentous injury in seven patients. Details of these additional injuries are summarised in Supplementary Table 1.

**Fig. 1:**
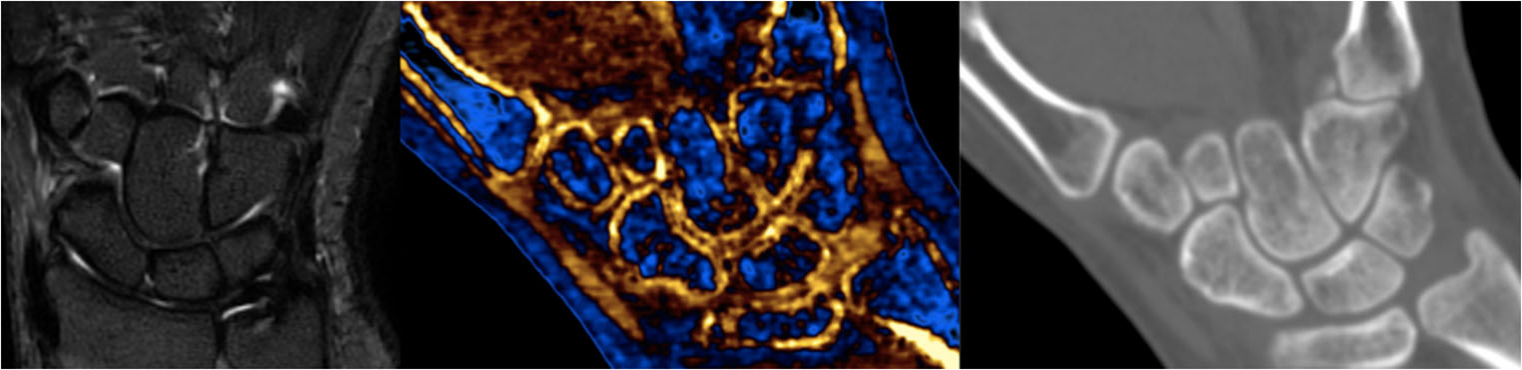
Normal scaphoid. No acute scaphoid bone marrow oedema on the STIR sequence of the MRI (left), DECT with calcium/water filter (middle) and no fracture on the CT component (right)

**Fig. 2:**
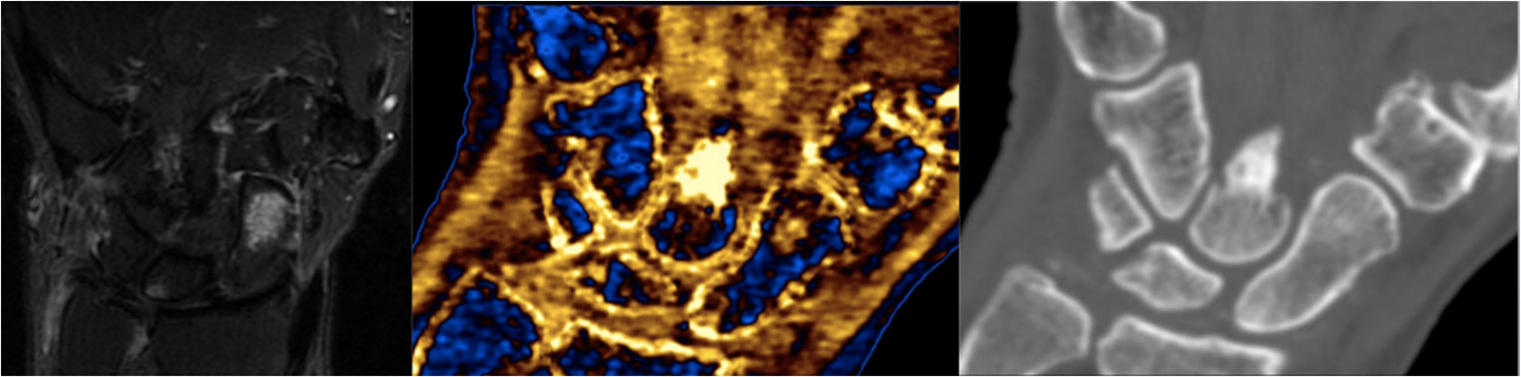
Focal and central acute scaphoid oedema. Both STIR sequence of the MRI (left) and DECT with calcium/water filter (middle) showed focal and central acute scaphoid oedema at the distal pole and waist of the scaphoid. The oedema is highlighted in gold on the DECT with calcium/water filter image. The CT component (right) did not show any fracture

**Fig. 3:**
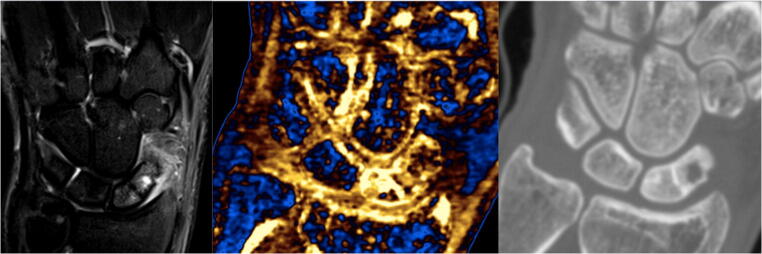
Diffuse acute scaphoid oedema. The STIR sequence of the MRI (left) and DECT with calcium/water filter (middle) showed diffuse scaphoid oedema. The oedema is highlighted in gold on the DECT with calcium/water filter image. The MRI also showed a distal pole fracture and a bone cyst in the proximal pole, which were also identifiable on the CT component (right)

**Fig. 4:**
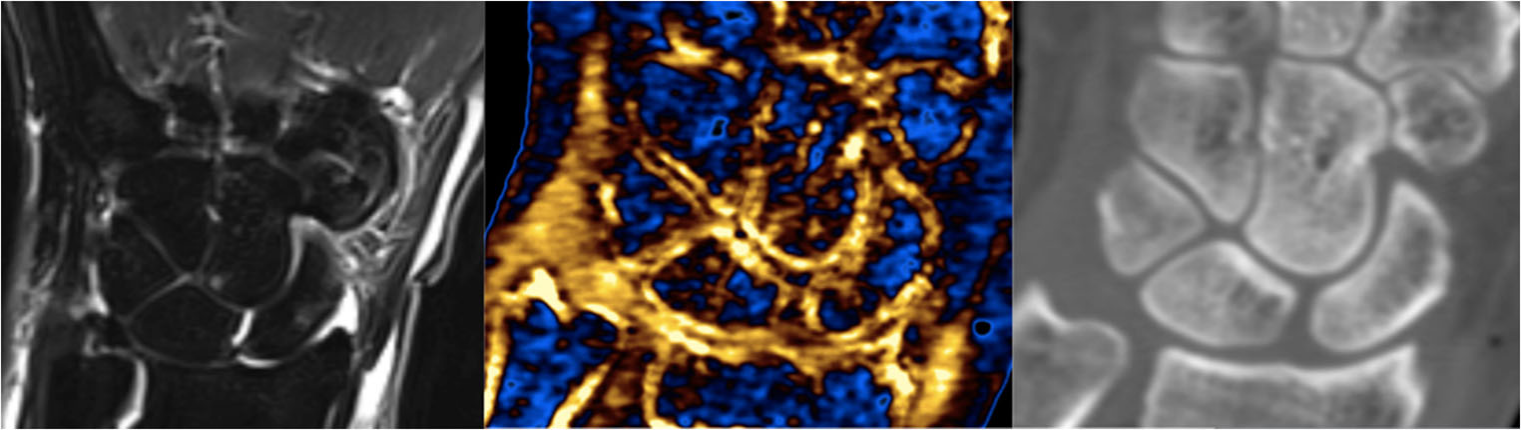
Mild acute scaphoid oedema. The STIR sequence of the MRI (left) showed a small peripheral area of oedema in the region of the waist of the scaphoid. This was difficult to detect with confidence on the DECT with calcium/water filter (middle) due to the ‘blooming’ artefact from the bony cortex. No fracture seen on the CT component (right)

On the DECT examination, reviewer A identified 13 of the 14 patients who had acute scaphoid oedema on their corresponding MRI (Fig. 1), and reviewer B identified 10 of the 14 patients. These 10 cases either demonstrated focal scaphoid oedema centrally (Fig. 2) or they had oedema throughout the scaphoid (Fig. 3). The four cases in which reviewer B did not identify any scaphoid oedema included the one patient who reviewer A also reported as no scaphoid injury. In these four cases, the MRI showed either mild or subcortical scaphoid oedema (Fig. 4). Areas of cortical bone were not suppressed in the calcium subtracted images and could not be differentiated from oedema.

The Friedman test was chosen to analyse for any statistically significant differences in scaphoid oedema detection between MRI and DECT (reviewers A and B). A patient with no reported scaphoid oedema was assigned a score of 0, and a patient with a reported presence of scaphoid oedema was assigned a score of 1. The results showed a *p* value of 0.61.

On the monochromatic component of the DECT examination, reviewer A identified all 6 fractures demonstrated on the MRI and detected an additional four fractures that were not demonstrated on the MRI. Reviewer B detected fractures in seven patients. Overall, scaphoid injury (oedema or fracture) was detected in all 14 MRI positive cases by reviewer A and 11 out of 14 MRI positive cases by reviewer B. Neither reviewer detected DECT scaphoid oedema in cases where there was no oedema demonstrated on the MRI.

The reviewers also identified non-scaphoid bony injury in six cases. This included a pisiform fracture not detected on MRI. Widening of the scapholunate interspace was demonstrated in the case where scapholunate injury was demonstrated on the MRI. The old ulnar styloid fracture did not show any bone marrow oedema on DECT.

## Discussion

Currently, there are a number of second-line diagnostic imaging investigations for occult fractures. Mallee et al (2015) conducted a Cochrane systematic review that compared the diagnostic accuracies of CT, MRI, and bone scan [11]. From 11 studies, 277 patients had CT, 221 had MRI, and 543 had a bone scan for clinically suspected scaphoid fractures; the sensitivity and specificity of CT were 0.72 and 0.99, of MRI 0.88 and 1.00, and of bone scan 0.99 and 0.86. The systematic review concluded that bone scan had the highest diagnostic accuracy, but it was the least preferred imaging examination due to its invasiveness and relatively low specificity, which would result in a larger number of unnecessary treatments. Indirect comparison between CT and MRI showed comparable diagnostic accuracies. The authors recommended either CT or MRI as the second-line imaging modality depending on availability. They also indicated that prospective studies involving CT and MRI in the same patient population would provide more valuable direct comparison between the diagnostic accuracies of two imaging options.

Subsequent to the above recommendations and our institution’s high dependence on MRI (one of the causes for the long waiting period of up to 51 days as shown in our study results), we proposed that DECT could be an alternative method for the diagnosis of scaphoid fracture including the detection of bone marrow oedema/bruising.

In our prospective study, we demonstrated that DECT could be used to identify moderate to severe focal and diffuse scaphoid bone marrow oedema. Apart from demonstrating acute bone marrow oedema, DECT was also able to delineate the small bony cortical disruptions to confirm the underlying fracture, which can be difficult to detect on MRI. Our results showed that DECT was limited in the detection of mild oedema seen on the MRI and/or oedema adjacent to the cortical surface due to lack of cortical bone suppression on the calcium subtracted images. Taking this limitation into account, statistically there was no significant difference in the detection of scaphoid bone marrow oedema between MRI and DECT. Scaphoid injury (oedema or fracture) was detected in all 14 MRI positive cases by reviewer A and 11 out of 14 MRI positive cases by reviewer B and the three cases where the injury was not detected by reviewer B were all treated conservatively indicating low clinical significance for failing to identify mild bone marrow oedema.

Although this is not the first study to use DECT to assess bone marrow oedema, it is currently the only published study that utilised the GE rapid voltage switching dual-energy CT (Revolution CT), and the water-calcium material decomposition images on using the Gemstone Spectral Imaging (GSI) viewer software for this purpose. The two recently published studies that investigated the diagnostic value of DECT in acute scaphoid and wrist injury both used the Siemens dual-source CT scanner and VNC reconstruction for the assessment of bone marrow oedema [9, 10]. Previous studies have also taken the same approach in the assessment of bone marrow oedema in the ankle, knee, hip, spine, and calcaneus [4–8]. The results of these studies concluded that DECT is an alternative to MRI for identifying bone marrow oedema including in the setting of acute scaphoid injuries.

Our study highlights a significant limitation of the water-calcium material decomposition technique in that it fails to differentiate between cortical bone and oedema, reducing its sensitivity for the detection of subcortical oedema. This is a known limitation of the DECT non-calcium technique [12, 13] and different methods have been suggested to overcome this such as altering the calcium suppression parameters [14] and using water-cortical bone material decomposition pairing [15].

One of the other limitations of this study is the time interval between the MRI and DECT and time interval between the first presentation to the emergency department and the DECT. It took up to 9 days between the MRI and DECT and up to 57 days from date of injury to DECT (although in this case both the CT and MRI were negative). The accuracy of bone marrow oedema detection related to the acute injury is likely to have been affected by the long waiting period. Following this study, we aim to implement DECT on the day of the presentation to improve the accuracy of acute bone marrow oedema detection. Furthermore, our study included only 20 patients and given the sample size, the statistical power of this study is limited. However, it does offer insight into bone marrow oedema detection using the GE DECT scanner and its analysis platform.

The acquisition time for a scaphoid DECT is a few seconds compared to our MRI protocol, which takes around 15 min. Additionally, MRI is a more expensive test and often less readily available after hours in an emergency setting. Using DECT as the second-line investigation following a negative radiograph would result in significantly improved resource utilisation.

CT scans are also more acceptable to patients as they are less likely to induce claustrophobia and are less noisy. However, this has to be balanced with the increased radiation dose associated with CT compared to MRI.

## Conclusion

DECT has the capacity to detect acute bone marrow oedema and identify subtle interruptions of scaphoid bony cortex to confirm a fracture. Its fast scanning time would be more suitable than MRI in the acute setting to exclude occult scaphoid fracture. A timely diagnosis would help avoid unnecessary treatments and follow-ups, as well as reduce the high reliance on MRI. However, further work is required on bone suppression algorithms to improve the sensitivity of subcortical bone marrow oedema detection.

## Supplementary information

ESM 1 (DOCX 82 kb)

## Abbreviations

CT: Computed tomography
DECT: Dual-energy computed tomography
FOOSH: Fall on an outstretched hand
MRI: Magnetic resonance imaging
VNC: Virtual non-calcium
