# Supplementary material for: Dual-energy CT in the diagnosis of occult acute scaphoid injury: a direct comparison with MRI
Source: Eur Radiol. 2020 Dec 19;31(6):3610–5. doi: 10.1007/s00330-020-07604-z (PMC8128856; doi:10.1007/s00330-020-07604-z)
Supplement: Supplementary file 1 — (DOCX 82 kb) [file 330_2020_7604_MOESM1_ESM.docx]

Supplementary Figure 1:


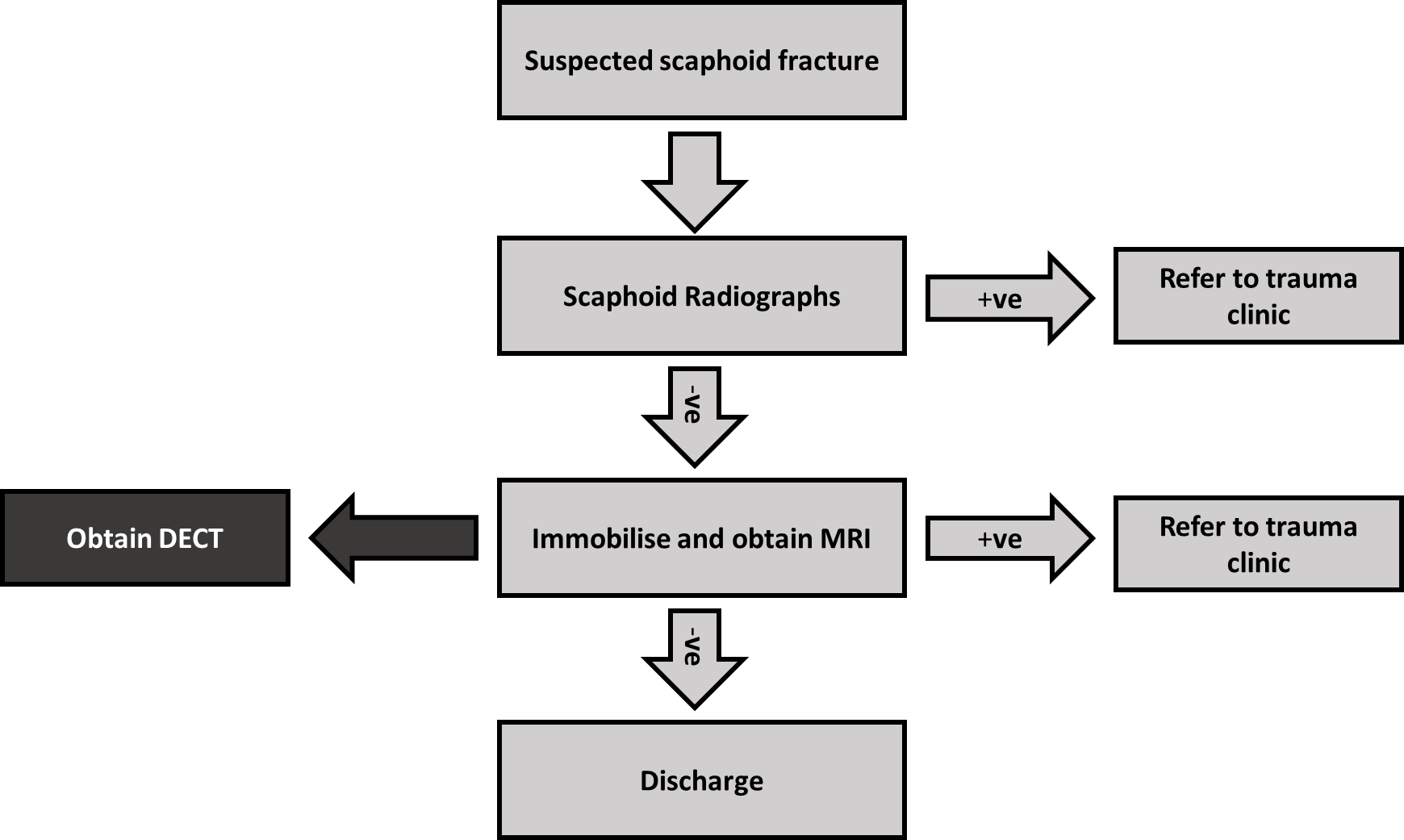


Supplementary figure 1: Patient flow for the study. The grey boxes indicate the usual clinical while the black box indicates the additional DECT investigation performed for the study

Supplementary Table 1: The table summarises the time from injury to hospital presentation and imaging, presence of scaphoid oedema and fractures on MRI and DECT, non scaphoid related MRI and CT findings and the treatment received by the patients.
